# Analysis for fractional‐order predator–prey model with uncertainty

**DOI:** 10.1049/iet-syb.2019.0055

**Published:** 2019-10-04

**Authors:** Samayan Narayanamoorthy, Dumitru Baleanu, Kalidas Thangapandi, Shyam Sanjeewa Nishantha Perera

**Affiliations:** ^1^ Department of Mathematics Bharathiar University Coimbatore Tamil Nadu India; ^2^ Department of Mathematics Cankaya University Balgat Ankara Turkey; ^3^ Institute of Space Sciences Bucharest Romania; ^4^ Research and Development Centre for Mathematical Modeling Faculty of Science University of Colombo Colombo Sri Lanka

**Keywords:** ecology, fuzzy set theory, predator‐prey systems, approximation theory, ecological model, fractional‐order predator–prey model, ecological systems, approximate solution, higher order term, fractional Euler method, uncertainty initial conditions, fuzzy initial conditions

## Abstract

Here, the authors analyse the fractional‐order predator–prey model with uncertainty, due to the vast applications in various ecological systems. The most of the ecological model do not have exact analytic solution, so they proposed a numerical technique for an approximate solution. In the proposed method, they have implemented the higher order term into the fractional Euler method to enhance the precise solution. Further, the present attempt is aimed to discuss the solutions of the FPPM with uncertainty (fuzzy) initial conditions. The initial conditions of the predator–prey model were taken as fuzzy initial conditions due to the fact that the ecological model highly depends on uncertain parameters such as growth/decay rate, climatic conditions, and chemical reactions. Finally, the numerical example manifest that the proposed method is authentic, applicable, easy to use from a computational viewpoint and the acquired outcomes are balanced with the existing method (HPM), which shows the efficiency of the proposed method.

## Nomenclature

**Table:** 

FDE	fuzzy differential equation
FEM	fractional Euler method
FFDE	fuzzy fractional differential equation
FPPM	fractional‐order predator–prey model
HPM	homotopy perturbation method
$\mu_{\tilde{A}}$	membership function
$\tilde{A}$	fuzzy set
*P*	densities of prey populations
*Q*	densities of predator populations
*a*	maximum per capita birth rate of prey species
*b*	intra‐specific competition of the prey species
*c*	intra‐specific between prey and predators
*e*	efficiency of ingested prey into new predators
*r*	membership grade
ρ	per capita death rate of the predator
R	real numbers
${}^{C}D^{n\beta }$	caputo fractional derivative
$J^{\beta }$	fractional integral
Γ(.)	gamma function

## 1 Introduction

The dynamical connection of predator and prey depict the predator–prey model which is utilised and described by the system of differential equations. Vito–Volterra (1860–1940) was a famous Italian mathematician who studied the populations of various species of fish in the Adriatic Sea in the period of First World War. In the meantime at the USA, the conditions considered by Volterra were determined autonomously by Alfred Lotka in 1925 [1] to portray a speculative synthetic response in which the compound fixations waver. The Lotka–Volterra models are the simplest predator–prey models, that are a couple of non‐linear differential equations, habitually used to portray the dynamics of bio and ecological systems with predator–prey, susceptible‐infectious [2], plant–herbivore, tumour cells (virus‐immune system) [3], parasite–host, resource–consumer interactions, and numerous other studies from diverse disciplines. The arthropod system [4], the killer whale pod in the wild [5], and prey refuge [6] are the few examples for the applications of predator–prey models. In the past two centuries, many researchers have made significant contributions to the field of predator–prey models [7, 8]. In general, predator–prey behaviour depends on various external factors, such as climatic/environment and biological factors. Hence, the outcomes of the predator–prey model significantly depend on these uncertainty behaviours of model parameters within the predator–prey framework which is an essential task. One approach to focus this issue is to discuss the outcomes in fuzzy environment.

The term fuzzy was introduced and developed in 1965 [9]. Genuine circumstances may have some ambiguous, inadequate, and deficient data about the factors and parameters because of a blunder in perception, test, and so on. These uncertainties might be demonstrated through fuzzy hypothesis. In last few decades, the attention of several researchers has done their work in the field of FDE (see in [10–13]).

Fractional calculus is the extension of ordinary calculus addition with fractional order. The purpose of this addition is to determine the maximum sustainable yield and exact description of real‐world phenomena. Fractional calculus was an immensely developed mathematical model of various fields such as biological systems, chemical problems, signal processing, control theory, and many real‐world problems [14–16]. From the point of the numerical solution, the FEM is derived for solving the differential equation of fractional order using a modified trapezoidal rule [17].

The combination of fuzzy and fractional calculus plays a vital role in the recent real‐world applications. Agarwal *et al.* [18] discussed the basic concept to obtain a solution of FFDE. Some results prove the existence and uniqueness of solutions of FFDE and its solution [19, 20]. In recent years, many researchers [21–26] have contributed to the theory of FFDE and its solution.

Over the last few years, one of the main attentions to the field of FFDE systems with their application is increased. In [27], the control and synchronisation of fractional‐order non‐linear systems have addressed by the fuzzy generalised predictive control for the fractional‐order brushless DC motor system and fractional‐order permanent magnet synchronous motor system. Agarwal *et al.* [28] addressed some representative results on fuzzy fractional differential equations, controllability, approximate controllability, optimal control, and optimal feedback control for several different kinds of fractional evolution equations. In [29], the existence of solutions for a class of fuzzy fractional differential systems with non‐local conditions under Caputo gH‐differentiability in generalised metric space in the sense of Perov was studied.

Moreover, Delavari *et al.* [30] investigated the adaptive fractional‐order blood glucose regulator model to control the blood glucose level of diabetes patients using sliding‐mode control. The human immunodeficiency virus (HIV) dynamic system model with some unknown parameters and unmeasurable CD8+T cell count were widely discussed and control of health status using the switching control strategy based on Lyapunov function theory by Ding and Wang [31]. The chaotic behaviour of love affairs model of fractional‐order system with fuzzy membership function as an external force was obtained by Huang and Bae [32]. In [33], a class of uncertain linear dynamical systems called fuzzy fractional linear dynamical systems were investigated and obtain the optimal control inputs of fuzzy fractional quadratic regulator problem under Granular fuzzy fractional derivatives.

Nevertheless, the predator–prey model is of the fractional order that was described and well explained by Petras in 2011 [34]. The fractional derivative involved in the predator–prey model is due to the fact that the aggregate properties of the parameters and processes of fragment memory management in biological systems. Therefore, many studies were focused to the FPPM and obtain significant results in [35]. The solution of the fractional‐order biological population model was obtained using the Adomian method in [36] and the FPPM was analysed and solved by HPM in [37–39]. The Caputo type fractional derivative system of non‐linear differential equation in the sense of FPPM was explained by Zhou and Xu in 2017 [40].

Therefore, there is an analysis need for research to improve the ecological model with uncertainty, so that it can be applied and evaluated by fractional predator–prey model which is the final motivation of this research article.

Motivated by the above discussions, this paper dealing with the most common biological system named as predator–prey model of fractional order with the fuzzy numbers. To predict the unknown parameters and initial conditions of the model, due to the fact that the ecological model highly depends on uncertain parameters such as growth/decay rate, climatic conditions, and chemical reactions. We have applied a newly proposed method to obtain the FPPM chaotic behaviour and time series solutions with changing the fractional order and uncertain parameter value.

The author contribution is given as follows:

(i) We proposed fractional modified Euler method which is improved from the FEM.
(ii) The proposed method is derived using the fuzzy generalised Taylor's expansion [41] and assumption of the generalised R–K method [42].
(iii) The main advantage of this proposed method is the higher efficiency for non‐linear systems and easily computable, which is validated through the numerical simulations that verify the effectiveness and superiority of this proposed method.

This paper is summarised as follows: First, basic definition and concepts are given. Second, the problem description and methods are discussed. Third, by considering the numerical examples, the effects of fuzzy FPPM and applicability and efficiency of the fractional modified Euler method are presented. Finally, the conclusion is drawn.

## 2 Preliminaries and problem formulation

Let $\tilde{A}$ be fuzzy number is defined by $\tilde{A}:R\to [0,1]$ have the following properties

(i) $\tilde{A}$ is upper semi‐continuous,
(ii) $\tilde{A}$ is fuzzy convex, $i.e.,\;\forall m,n\in R,\;\rho \in [0,1],\;\tilde{A}(\rho m+(1-\rho )n)\geq \min\{\tilde{A}(m),\tilde{A}(n)\},$
(iii) $\tilde{A}$ is normal, $i.e.,\;\exists m_{0}\in R$ for which $\tilde{A}(m_{0})=1$,
(iv) $\text{supp}\;\tilde{A}=\{m\in R|A(m)>0\}$ is the support of the $\tilde{A}$, and its closure $\text{cl}(\text{supp}\;\tilde{A})$ is compact.

A triangular fuzzy number $\tilde{A}=(m,n,o)$ is a convex normalised fuzzy set $\tilde{A}$ of the real line R. Then, the membership function $\mu_{\tilde{A}}$ of $\tilde{A}$ is addressed by,

(1) μA~(φ)=0,0000φ≤mφ−mn−m,ifm≤φ≤no−φo−n,ifn≤φ≤o0,0000φ≥o

and its ordered pair of functions through the *r* ‐cut form is $\left[\underline{A}(r),\bar{A}(r)]=[(n-m)r+m,\;o-(o-n)r\right]$ where r∈[0,1].

**Fig. 1:**
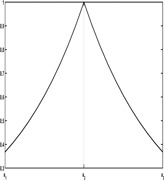
Shape of the exponential triangular number

A fuzzy set $\tilde{A}=(m,n,o)$ is an exponential fuzzy number. Its membership function $\nu_{\tilde{A}}$ of $\tilde{A}$ is,

(2) νA~(φ)=exp−(n−φ)n−m,ifm≤φ≤nexp−(φ−n)o−n,ifn≤φ≤o0,00000otherwise

and its reference functions through the *r* ‐cut form is $\left[\underline{A}(r),\bar{A}(r)]=[n+(n-m)\log r,\;n-(o-n)\log r\right]$ where r∈[0,1] (Fig. 1).

The Riemann–Liouville fractional integral operator $\left(J^{\beta }\right)$ of order β≥0, of function $u\in C_{\eta },\eta \geq -1$

(3) $$J^{\beta }u(t)=\frac{1}{\Gamma (\beta )}\int_{0}^{t}(t-\tau ){\;}^{\left(\beta -1\right)}u(\tau )d\tau ,\;\;(\beta >0)$$

(4) $$J^{0}u(t)=u(t)$$

The fractional derivative ${(}^{C}D^{\beta })$ of u(t) in the Caputo sense is as follows

(5) DCDβu(t)=1Γ(n−β)∫t0(t−τ)n−β−1u(n)dτ

for $n-1<\beta \leq n,n\in N,u\in C_{\left(-1\right)}^{n}$.

(see in, [43]) Suppose that DCDnβu(t)∈C(0,a] for n=0,1,…,j+1, where 0<β≤1. Then, generalised Taylor's formula is defined as

(6) u(t)=∑n=0jtnβΓ(nβ+1)DCDnβ(0+)+(CD(j+1)β)u(ζ)Γ((j+1)β+1)t(j+1)β

with 0≤ζ≤t,∀t∈(0,a].

. Using the relation

$$(J^{j\beta }{\;}^{C}D^{j\beta }u)t-(J^{(j+1)\beta }{\;}^{C}D^{(j+1)\beta }u)t=\frac{{\left(t-a\right)}^{j\beta }}{\Gamma (j\beta +1)}{(}^{C}D^{j\beta }u)a$$

we have

(7) $$\sum_{n=0}^{j}(J^{n\beta }{\;}^{C}D^{n\beta }u)t-(J^{(n+1)\beta }{\;}^{C}D^{(n+1)\beta }u)t=\sum_{n=0}^{j}\frac{{\left(t-a\right)}^{n\beta }}{\Gamma (n\beta +1)}{(}^{C}D^{n\beta }u)a$$

that is

(8) $$u(t)-(J^{(j+1)\beta }{\;}^{C}D^{(j+1)\beta }u)t=\sum_{n=0}^{j}\frac{{\left(t-a\right)}^{n\beta }}{\Gamma (n\beta +1)}{(}^{C}D^{n\beta }u)a$$

Applying the integral mean value theorem yields

(9) $$(J^{(j+1)\beta }{\;}^{C}D^{(j+1)\beta }u)t=\frac{{(}^{C}D^{(j+1)\beta }u)\zeta }{\Gamma ((j+1)\beta +1)}t^{(j+1)\beta }$$

Using (8) and (9), (6) is obtained. This completes the proof.□

## 3 Problem description and methods

Now, we discuss the FPPM to frame the system of fractional differential equation as predator–prey model as follows:

(10) DCDβ1u(t)=f1(t,u,v)

(11) DCDβ2v(t)=f2(t,u,v)

(12) $$u(t_{0})=u_{0}$$

(13) $$v(t_{0})=v_{0}$$

where $f_{1}:[t_{0},T]\times R\to R$ and $f_{2}:[t_{0},T]\times R\to R$ are real‐valued function, $u_{0},v_{0}\in R$, and $\beta_{1},\beta_{2}\in (0,1]$. Let $\beta_{1}=\beta_{2}=1$ be the explicit form of ordinary system of differential equation.

Here, we have taken that the initial value is a triangular fuzzy number as

(14) $$\tilde{u}(t_{0})=(a_{1},\;b_{1},\;c_{1})$$

(15) $$\tilde{v}(t_{0})=(a_{2},\;b_{2},\;c_{2})$$

then (10) and (11) read as

(16) $${}^{C}D^{\beta_{1}}\tilde{u}(t)=f_{1}(t,\tilde{u},\tilde{v})$$

(17) $${}^{C}D^{\beta_{2}}\tilde{v}(t)=f_{2}(t,\tilde{u},\tilde{v})$$

with fuzzy initial conditions of (14) and (15). For the most part, the solution of (16) and (17) may not be found analytically. Therefore, a numerical analysis need to be used. FEM under the Caputo fractional derivative implemented by Odibat and Momani in 2008 [17].

Here, we extend the FEM to solve the system of FFDE.

(18) $$\begin{array}{c} {\tilde{u}}_{j+1}(t;r)={\tilde{u}}_{j}+\frac{h^{\beta_{1}}}{\Gamma (\beta_{1}+1)}f_{1}(t,{\tilde{u}}_{j},{\tilde{v}}_{j})\\ {\tilde{v}}_{j+1}(t;r)={\tilde{v}}_{j}+\frac{h^{\beta_{2}}}{\Gamma (\beta_{2}+1)}f_{2}(t,{\tilde{u}}_{j},{\tilde{v}}_{j}) \end{array}$$

However, in order to improve the accuracy of an approximate solution of system FFDE, incorporating the fractional Taylor series formula [43] with generalised R–K method [42], the following fractional modified Euler method is obtained.

(19) $$\begin{array}{c} {\tilde{u}}_{i+1}(t;r)={\tilde{u}}_{i}+\Delta {\tilde{u}}_{i}\\ {\tilde{v}}_{i+1}(t;r)={\tilde{v}}_{i}+\Delta {\tilde{v}}_{i} \end{array}$$

(20) $$\begin{array}{rl} & \Delta {\tilde{u}}_{i}=\frac{h^{\beta_{1}}}{\Gamma (\beta_{1}+1)}f_{1}(t+X1,\;{\tilde{u}}_{i}+X1f_{1}(t,{\tilde{u}}_{i},{\tilde{v}}_{i}),\\ & \;{\tilde{v}}_{i}+X1f_{1}(t,{\tilde{u}}_{i},{\tilde{v}}_{i})) \end{array}$$

(21) $$\begin{array}{rl} & \Delta {\tilde{v}}_{i}=\frac{h^{\beta_{2}}}{\Gamma (\beta_{2}+1)}f_{2}(t+Y1,\;{\tilde{u}}_{i}+Y1f_{2}(t,{\tilde{u}}_{i},{\tilde{v}}_{i}),\\ & \;{\tilde{v}}_{i}+Y1f_{2}(t,{\tilde{u}}_{i},{\tilde{v}}_{i})) \end{array}$$

where

$$X1=\frac{\Gamma (\beta_{1}+1)h^{\beta_{1}}}{\Gamma (2\beta_{1}+1)},\;\;Y1=\frac{\Gamma (\beta_{2}+1)h^{\beta_{2}}}{\Gamma (2\beta_{2}+1)}$$

## 4 Numerical simulation

Consider the following FPPM [39]

(22) DCDtβ1u~(t)=w(t)u~(t)−x(t)u~(t)v~(t)DCDtβ2v~(t)=y(t)u~(t)v~(t)−z(t)v~(t)

with fuzzy initial conditions

(23) μu~(0;r)=0,00000t≤1.2t−1.21.3−1.2,if1.2≤t≤1.31.4−t1.4−1.3,if1.3≤t≤1.40,00000t≥1.4

(24) μv~(0;r)=0,00000t≤0.5t−0.50.6−0.5,if0.5≤t≤0.60.7−t0.7−0.6,if0.6≤t≤0.70,00000t≥0.7

and its parametric form becomes

$$\tilde{u}(0;r)=[r(1.3-1.2)+1.2,\;\;1.4-(1.4-1.3)r]$$

$$\tilde{v}(0;r)=[r(0.6-0.5)+0.5,\;\;0.7-(0.7-0.6)r]$$

(25) νu~(0;r)=exp−(1.3−t)1.3−1.2,if1.2≤t≤1.3exp−(t−1.3)1.4−1.3,if1.3≤t≤1.40,00000otherwise

(26) νv~(0;r)=exp−(0.6−t)0.6−0.5,if0.5≤t≤0.6exp−(t−0.6)0.7−0.6,if0.6≤φ≤0.70,00000otherwise

and its reference functions in ordered pair is as follows

$$\tilde{u}(0;r)=[\;1.3+(1.3-1.2)\log r,\;\;1.3-(1.4-1.3)\log r\;]$$

$$\tilde{v}(0;r)=[\;0.6+(0.6-0.5)\log r,\;\;0.6-(0.7-0.6)\log r\;]$$

Here, we consider two cases of FPPM such as

*Case 1:* w(t)=t,x(t)=1,y(t)=1,z(t)=t
*Case 2:* w(t)=1,x(t)=t,y(t)=t,z(t)=1

An approximate solution of FPPM is given below

$$\tilde{u}(t;r)=[\underline{u}(t;r),\;\;\bar{u}(t;r)\;]\;\;\text{and}$$

$$\tilde{v}(t;r)=[\underline{v}(t;r),\;\;\bar{v}(t;r)]$$

By applying required functions and fuzzy initial conditions into the FEM (18) and fractional modified Euler formula (19), we have

(27) $$\begin{array}{rl} & {\underline{u}}_{1}(t;r)={\underline{u}}_{0}(t;r)+\frac{h^{\beta_{1}}}{\Gamma (\beta_{1}+1)}f_{1}(t,{\underline{u}}_{0},{\underline{v}}_{0})\\ & {\bar{u}}_{1}(t;r)={\bar{u}}_{0}(t;r)+\frac{h^{\beta_{1}}}{\Gamma (\beta_{1}+1)}f_{1}(t,{\bar{u}}_{0},{\bar{v}}_{0})\\ & {\underline{v}}_{1}(t;r)={\underline{v}}_{0}(t;r)+\frac{h^{\beta_{2}}}{\Gamma (\beta_{2}+1)}f_{2}(t,{\underline{u}}_{0},{\underline{v}}_{0})\\ & {\bar{v}}_{1}(t;r)={\bar{v}}_{0}(t;r)+\frac{h^{\beta_{2}}}{\Gamma (\beta_{2}+1)}f_{2}(t,{\bar{u}}_{0},{\bar{v}}_{0}) \end{array}$$

(28) $$\begin{array}{rl} & {\underline{u}}_{1}(t;r)={\underline{u}}_{0}(t;r)+\Delta {\underline{u}}_{0}\\ & {\bar{u}}_{1}(t;r)={\bar{u}}_{0}(t;r)+\Delta {\bar{u}}_{0}\\ & {\underline{v}}_{1}(t;r)={\underline{v}}_{0}(t;r)+\Delta {\underline{v}}_{0}\\ & {\bar{v}}_{1}(t;r)={\bar{v}}_{0}(t;r)+\Delta {\bar{v}}_{0} \end{array}$$

(29) $$\begin{array}{rl} & {\underline{u}}_{2}(t;r)={\underline{u}}_{1}(t;r)+\frac{h^{\beta_{1}}}{\Gamma (\beta_{1}+1)}f_{1}(t,{\underline{u}}_{1},{\underline{v}}_{1})\\ & {\bar{u}}_{2}(t;r)={\bar{u}}_{1}(t;r)+\frac{h^{\beta_{1}}}{\Gamma (\beta_{1}+1)}f_{1}(t,{\bar{u}}_{1},{\bar{v}}_{1})\\ & {\underline{v}}_{2}(t;r)={\underline{v}}_{1}(t;r)+\frac{h^{\beta_{2}}}{\Gamma (\beta_{2}+1)}f_{2}(t,{\underline{u}}_{1},{\underline{v}}_{1})\\ & {\bar{v}}_{2}(t;r)={\bar{v}}_{1}(t;r)+\frac{h^{\beta_{2}}}{\Gamma (\beta_{2}+1)}f_{2}(t,{\bar{u}}_{1},{\bar{v}}_{1}) \end{array}$$

(30) $$\begin{array}{c} {\underline{u}}_{2}(t;r)={\underline{u}}_{1}(t;r)+\Delta {\underline{u}}_{1}\\ {\bar{u}}_{2}(t;r)={\bar{u}}_{1}(t;r)+\Delta {\bar{u}}_{1}\\ {\underline{v}}_{2}(t;r)={\underline{v}}_{1}(t;r)+\Delta {\underline{v}}_{1}\\ {\bar{v}}_{2}(t;r)={\bar{v}}_{1}(t;r)+\Delta {\bar{v}}_{1} \end{array}$$

and so on.

Similarly, we can obtain the succeeding points using both proposed methods. The solution to two cases of FPPM using triangular and exponential fuzzy numbers is given in the pictorial representation.

Consider the following Lotka–Volterra FPPM [44]

(31) DCDTβ1P(T)=P(T)a−bP(T)−cQ(T)DCDTβ2Q(T)=Q(T)−ρ+ceP(T)

The above system is transformed by reducing the parameters and the non‐dimensionless form as follows

(32) DCDtβ1u~(t)=u~(t)l−ku~(t)−v~(t)DCDtβ2v~(t)=v~(t)−1+αu~(t)

where $\tilde{u}(t)=\left(\text{ea}/\rho \right)P(T)$, $\tilde{v}(t)=\left(c/\rho \right)Q(T),t=\rho T$, $l=\left(a/\rho \right)$, $k=\left(b/\text{ae}\right)$, $\alpha =\left(c/a\right)$ with fuzzy initial conditions

(33) μu~(0;r)=0,00000t≤0.15t−0.150.25−0.15,if0.15≤t≤0.250.35−t0.35−0.25,if0.25≤t≤0.350,00000t≥0.35

(34) μv~(0;r)=0,00000t≤0.1t−0.10.2−0.1,if0.1≤t≤0.20.3−t0.3−0.2,if0.2≤t≤0.30,00000t≥0.3

and its parametric form becomes

$$\tilde{u}(0;r)=[r(0.25-0.15)+0.15,\;\;0.35-(0.35-0.25)r]$$

$$\tilde{v}(0;r)=[r(0.2-0.1)+0.1,\;\;0.3-(0.3-0.2)r]$$

(35) νu~(0;r)=exp−(0.25−t)0.25−0.15,if0.15≤t≤0.25exp−(t−0.25)0.35−0.25,if0.25≤t≤0.350,00000otherwise

(36) νv~(0;r)=exp−(0.2−t)0.2−0.1,if0.1≤t≤0.2exp−(t−0.2)0.3−0.2,if0.2≤t≤0.30,00000otherwise

and its reference functions in ordered pair is as follows:

$$\tilde{u}(0;r)=[0.25+(0.25-0.15)\log r,\;\;0.25-(0.35-0.25)\log r]$$

$$\tilde{v}(0;r)=[0.2+(0.2-0.1)\log r,\;\;0.2-(0.3-0.2)\log r]$$

Now, we obtain the numerical solution by successive iterative procedure via proposed method and the behaviour of the Lotka–Volterra FPPM are given in graphical form.

## 5 Result and discussion

Now, we analyse the numerical simulations of FPPM using fractional modified Euler method. According to the given FPPM, many parameters are enrolled to depict the various models. As such, the two models are taken here to perform a numerical analysis.

**Fig. 2:**
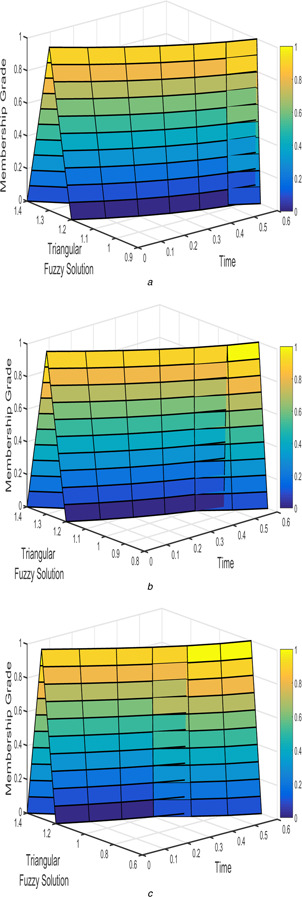
*Triangular fuzzy solution* $\tilde{u}(t;r)$ *of FPPM* (22) *of Case 1 for* ***(a)*** $\beta_{1}=\beta_{2}=1$, ***(b)*** $\beta_{1}=\beta_{2}=9/10$, ***(c)*** $\beta_{1}=\beta_{2}=4/5$

**Fig. 3:**
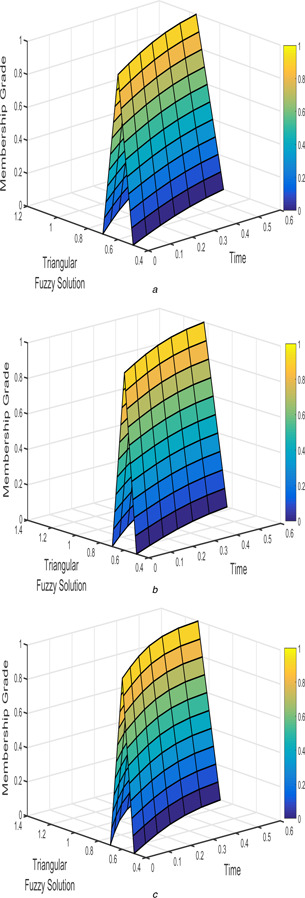
*Triangular fuzzy solution* $\tilde{v}(t;r)$ *of FPPM* (22) *of Case 1 for* ***(a)*** $\beta_{1}=\beta_{2}=1$, ***(b)*** $\beta_{1}=\beta_{2}=9/10$, ***(c)*** $\beta_{1}=\beta_{2}=4/5$

**Fig. 4:**
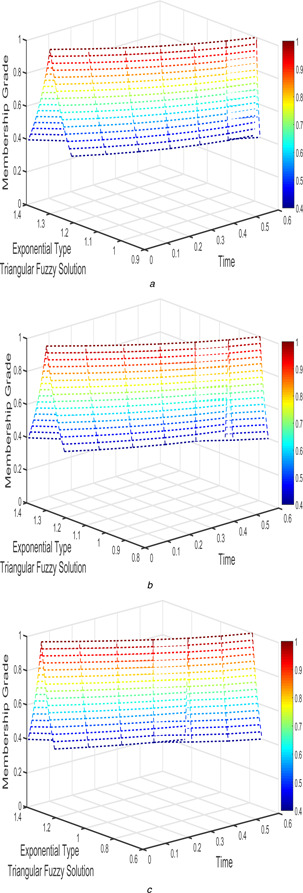
*Exponential triangular fuzzy solution* $\tilde{u}(t;r)$ *of FPPM* (22) *of Case 1 for* ***(a)*** $\beta_{1}=\beta_{2}=1$, ***(b)*** $\beta_{1}=\beta_{2}=9/10$, ***(c)*** $\beta_{1}=\beta_{2}=4/5$

**Fig. 5:**
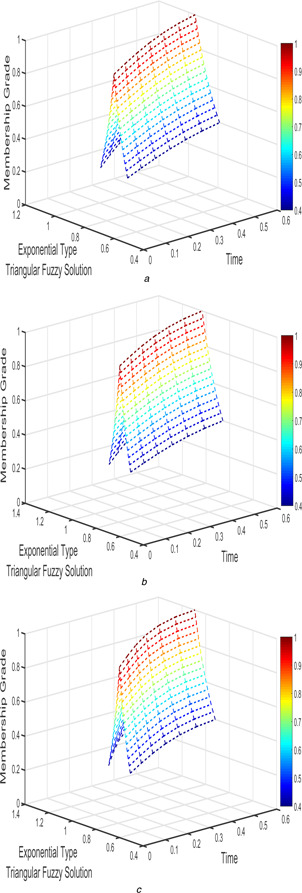
*Exponential triangular fuzzy solution* $\tilde{v}(t;r)$ *of FPPM* (22) *of Case 1 for* ***(a)*** $\beta_{1}=\beta_{2}=1$, ***(b)*** $\beta_{1}=\beta_{2}=9/10$, ***(c)*** $\beta_{1}=\beta_{2}=4/5$

The interval triangular fuzzy solution for model 1 of case 1 with different arbitrary derivatives such as $\beta_{1}=\beta_{2}=1$, $\beta_{1}=\beta_{2}=9/10$, and $\beta_{1}=\beta_{2}=4/5$ are plotted in Figs. 2 and 3. Similarly, the exponential triangular interval fuzzy solution is presented in Figs. 4 and 5.

**Fig. 6:**
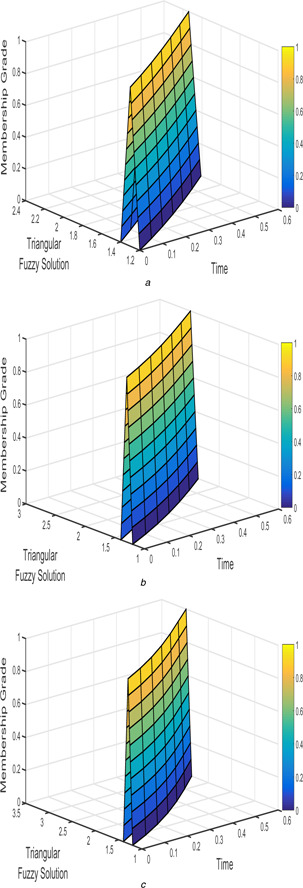
*Triangular fuzzy solution* $\tilde{u}(t;r)$ *of FPPM* (22) *of Case 2 for* ***(a)*** $\beta_{1}=\beta_{2}=1$, ***(b)*** $\beta_{1}=\beta_{2}=9/10$, ***(c)*** $\beta_{1}=\beta_{2}=4/5$

**Fig. 7:**
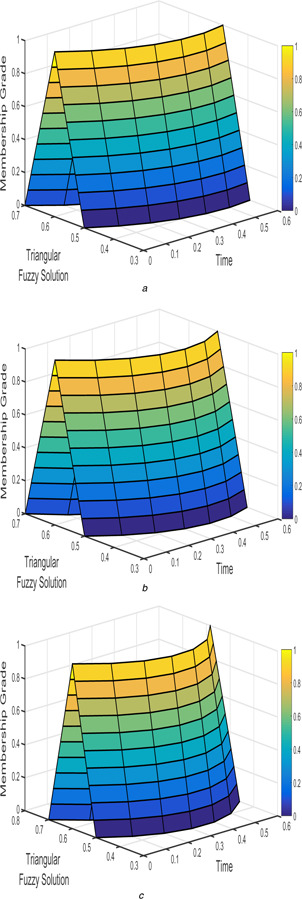
*Triangular fuzzy solution* $\tilde{v}(t;r)$ *of FPPM* (22) *of Case 2 for* ***(a)*** $\beta_{1}=\beta_{2}=1$, ***(b)*** $\beta_{1}=\beta_{2}=9/10$, ***(c)*** $\beta_{1}=\beta_{2}=4/5$

**Fig. 8:**
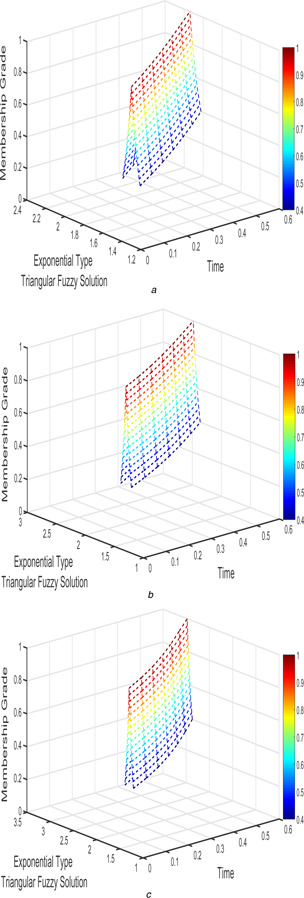
*Exponential triangular fuzzy solution* $\tilde{u}(t;r)$ *of FPPM* (22) *of Case 2 for* ***(a)*** $\beta_{1}=\beta_{2}=1$, ***(b)*** $\beta_{1}=\beta_{2}=9/10$, ***(c)*** $\beta_{1}=\beta_{2}=4/5$

**Fig. 9:**
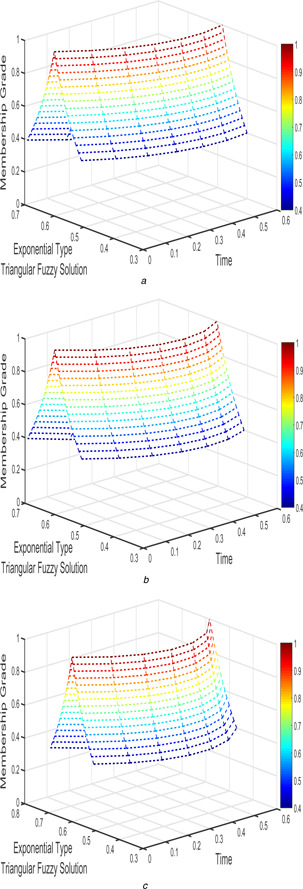
*Exponential triangular fuzzy solution* $\tilde{v}(t;r)$ *of FPPM* (22) *of Case 2 for* ***(a)*** $\beta_{1}=\beta_{2}=1$, ***(b)*** $\beta_{1}=\beta_{2}=9/10$, ***(c)*** $\beta_{1}=\beta_{2}=4/5$

For Case 2, several of fractional order which has applied for FPPM and its interval‐valued solutions are shown in Figs. 6, 7, 8–9.

**Fig. 10:**
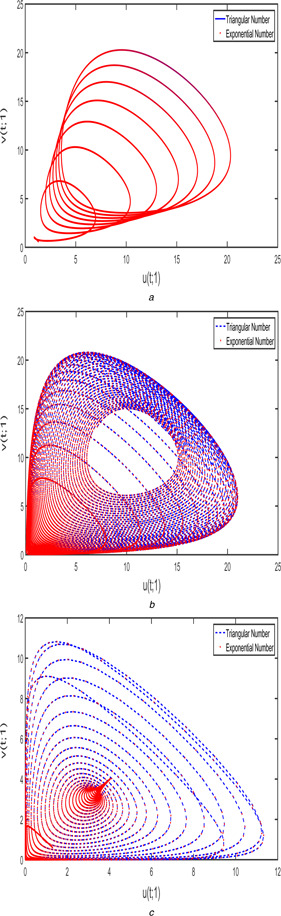
*Phase portrait for FPPM* (22) *of Case 1 with different fractional orders* ***(a)*** $\beta_{1}=\beta_{2}=1$, ***(b)*** $\beta_{1}=\beta_{2}=3/4$, ***(c)*** $\beta_{1}=\beta_{2}=1/2$

**Fig. 11:**
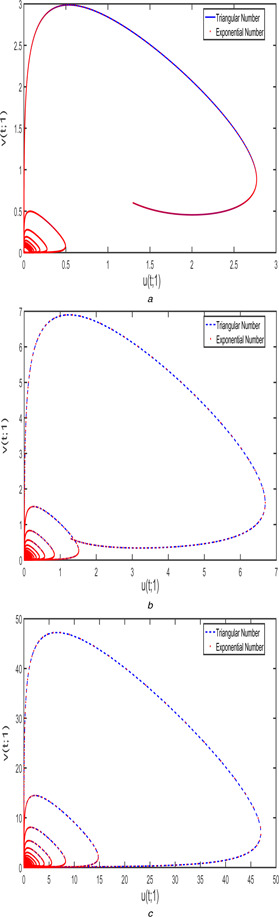
*Phase portrait for FPPM* (22) *of Case 2 with different fractional orders* ***(a)*** $\beta_{1}=\beta_{2}=1$, ***(b)*** $\beta_{1}=\beta_{2}=3/4$, ***(c)*** $\beta_{1}=\beta_{2}=1/2$

Figs. 10 and 11 display the phase portrait view of FPPM (22) corresponding to case 1 and case 2 with different fractional‐order $(a)\;\beta_{1}=\beta_{2}=1$, $(b)\;\beta_{1}=\beta_{2}=3/4$, and $(c)\;\beta_{1}=\beta_{2}=1/2$. This chaotic behaviour is compared according to the triangular and exponential fuzzy number.

**Fig. 12:**
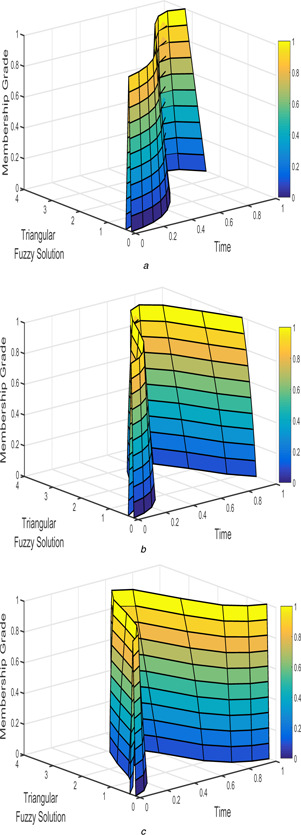
*Triangular fuzzy solution* $\tilde{u}(t;r)$ *of FPPM* (31) *for* ***(a)*** $\beta_{1}=\beta_{2}=1$, ***(b)*** $\beta_{1}=\beta_{2}=9/10$, ***(c)*** $\beta_{1}=\beta_{2}=4/5$

**Fig. 13:**
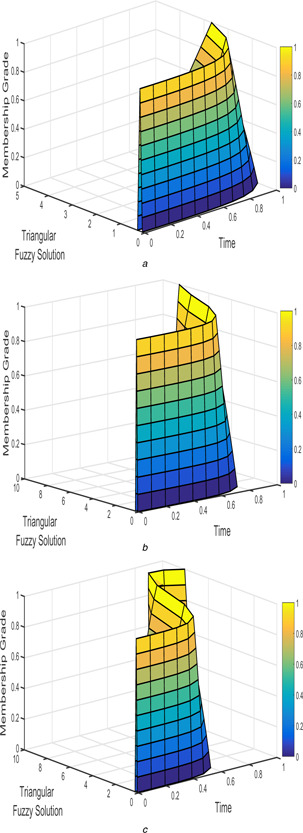
*Triangular fuzzy solution* $\tilde{v}(t;r)$ *of FPPM* (31) *for* ***(a)*** $\beta_{1}=\beta_{2}=1$, ***(b)*** $\beta_{1}=\beta_{2}=9/10$, ***(c)*** $\beta_{1}=\beta_{2}=4/5$

**Fig. 14:**
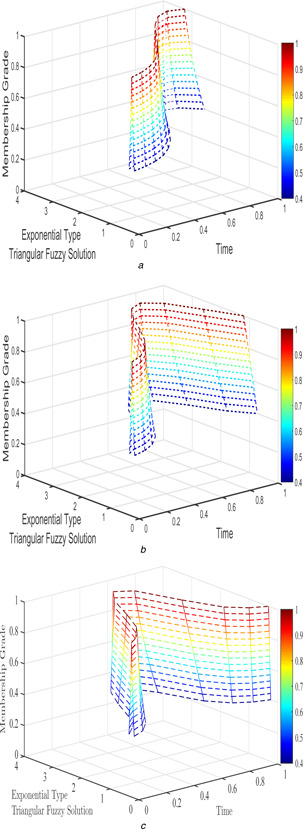
*Exponential triangular fuzzy solution* $\tilde{u}(t;r)$ *of FPPM* (31) *for* ***(a)*** $\beta_{1}=\beta_{2}=1$, ***(b)*** $\beta_{1}=\beta_{2}=9/10$, ***(c)*** $\beta_{1}=\beta_{2}=4/5$

**Fig. 15:**
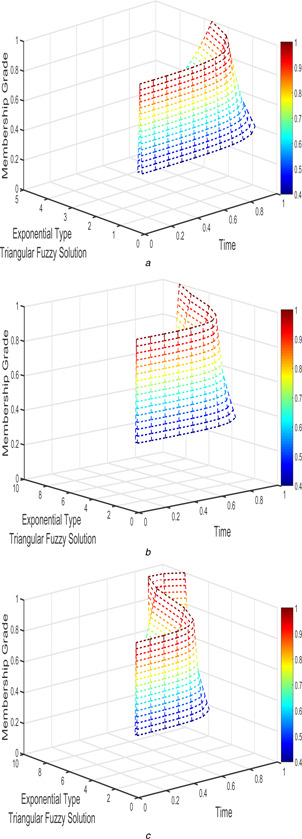
*Exponential triangular fuzzy solution* $\tilde{v}(t;r)$ *of FPPM* (31) *for* ***(a)*** $\beta_{1}=\beta_{2}=1$, ***(b)*** $\beta_{1}=\beta_{2}=9/10$, ***(c)*** $\beta_{1}=\beta_{2}=4/5$

The interval triangular and exponential fuzzy solution of model 2 (l=5;k=1;α=2) for various fractional order taken as $\beta_{1}=\beta_{2}=1$, $\beta_{1}=\beta_{2}=9/10$, and $\beta_{1}=\beta_{2}=4/5$ are shown in Figs. 12, 13, 14–15.

**Fig. 16:**
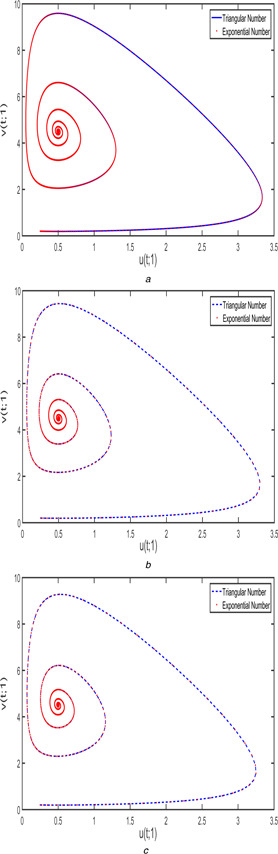
*Phase portrait for FPPM* (22) *with different fractional orders* ***(a)*** $\beta_{1}=\beta_{2}=1$, ***(b)*** $\beta_{1}=\beta_{2}=3/4$, ***(c)*** $\beta_{1}=\beta_{2}=1/2$

Fig. 16 exhibits the chaotic behaviour of FPPM (32) with various fractional orders such as $(a)\;\beta_{1}=\beta_{2}=1$, $(b)\;\beta_{1}=\beta_{2}=3/4$, and $(c)\;\beta_{1}=\beta_{2}=1/2$. The blue‐dashed line indicates the phase diagram using triangular membership function and red‐doted mentions the exponential membership function with *r* ‐level as 1. From the above discussion, we may conclude that the triangular and exponential membership functions are almost same in the solution space with r=1 and the changes in left and right reference functions clearly indicate the properties of triangular and exponential fuzzy number.

**Fig. 17:**
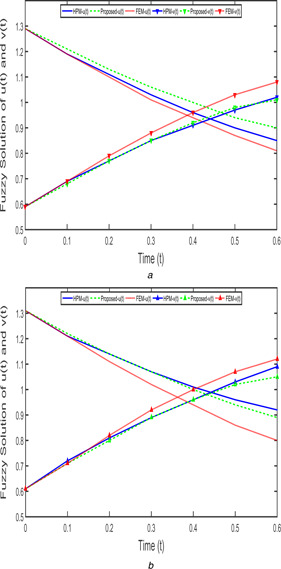
*Fuzzy solution of FPPM* (22) *for Case 1 with* $\beta_{1}=\beta_{2}=9/10,h=0.1$ ***(a)*** $\underline{u}(t;0.9)\&\underline{v}(t;0.9)$, ***(b)*** $\bar{u}(t;0.9)\&\bar{v}(t;0.9)$

**Fig. 18:**
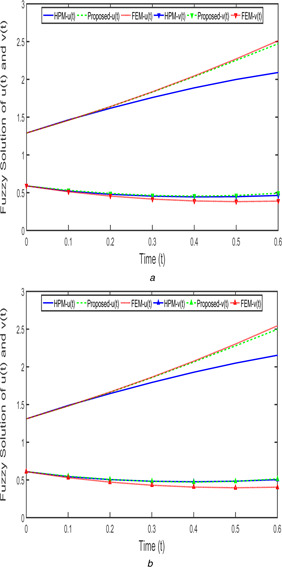
*Fuzzy solution of FPPM* (22) *for Case 2 with* $\beta_{1}=\beta_{2}=9/10,h=0.1$ ***(a)*** $\underline{u}(t;0.9)\&\underline{v}(t;0.9)$, ***(b)*** $\bar{u}(t;0.9)\&\bar{v}(t;0.9)$

Fig. 17 *a* depict that the solutions of FPPM (22) of case 1 in the fractional‐order $\left(\beta_{1}=\beta_{2}=9/10\right)$ with increasing uncertainty parameter (r=0.9) which are obtained by FEM, the proposed method, and HPM. Using non‐increasing fuzzy parameter (r=0.9), the fractional‐order $\left(\beta_{1}=\beta_{2}=9/10\right)$ predator–prey model of case 1 solutions behaviour are done via FEM, HPM, and proposed method are shown in Fig. 17 *b*. By using FEM and the proposed method, taken the step size as h=0.1. Similarly, the time series solution of FPPM (22) of case 2 with increasing and decreasing fuzzy parameters (r=0.9) are shown in Figs. 18 *a* and Fig. 18 *b*.

From Figs. 17 *a*, *b* and 18 *a*, *b*, the obtained proposed method solution is improved from FEM and also merely coincide with HPM are depicted and one can see that the prey population decreases and predator population increases with respect to time *t*.

## 6 Conclusion

The focus theme of this work is to construct an approximate solution of the system of FFDE. The aim has been achieved by using fractional modified Euler method. In general, the proposed method can be implemented to solve the system of non‐linear and linear problems in FFDE. Predator–prey model is one of the most important applications of the system of differential equations. Therefore, we considered a numerical example as a FPPM with fuzzy conditions. Numerical results obtained from the proposed method show the applicability, accuracy, and efficiency, compared to the results obtained from existing methods. The primary purpose of the proposed method is easily computable components compared to semi‐analytical methods.

By incorporating with higher order terms from generalised Taylor series formula, the accuracy of the solution can be improved. Therefore, the accuracy of the proposed method can be improved extending the model by including higher order terms.

## Author contributions

All authors have contributed equally in the idea of the article. Also all authors have validated and approved the final manuscript.
